# Professional Procedure and Panel Patients

**Published:** 1915-06-12

**Authors:** 


### The Hospital

The Workers' Newspaper of Administrative Medicine and Institutional Life, Administration, National Insurance and Health.

No. 1512, Vol. LVIII. SATURDAY, JUNE 12, 1915. PRICE ONE PENNY

### PROFESSIONAL PROCEDURE AND PANEL PATIENTS.

If the National Insurance Act has not exactly brought a sword it has certainly not brought peace. The points of contact which it decrees between the medical profession on the one hand, and the insurance committees, friendly societies, and pharmacists on the other, mean inevitably some measure of friction, and the friction appears to be a growing quantity. The disputes which thus arise are just now largely concealed from public attention by the urgency of external affairs, but readers of the local provincial newspapers are painfully aware both of their numbers and of the tempers which they excite. Discussions are constantly reported in which the actions of the medical profession, or of one or other of its individual members, are challenged or censured. The criticism is not always well-informed, nor is it always just; and it is made very frequently in circumstances which do not permit either of explanation or of defence. All the same it is well to remember that in consequence of existing conditions professional procedure is apt to be forced into the public gaze, and that it is specially necessary to avoid whatever may savour of departure from the highest standards. The good name of the profession is in some degree committed into the hands of each of its individual members, and though it would be folly and weakness to yield to ignorant clamour, care ought to be taken to avoid even the appearance of evil.

It is rightly the boast of the profession that its first consideration is always the welfare of the patient, and that no question of formal etiquette or of personal *amour propre* ought to be allowed to interfere with this aim. The public does not always understand the best means of securing such a result and is sometimes ready to ascribe to false pride measures which are essential to the patient's interest. There is, no doubt, need for education in this direction, but the process ought to be conducted in such a manner that no harm is inflicted on the individual sufferer. These principles, we think, are illustrated in a dispute which has recently been the subject of a somewhat formal inquiry. A medical practitioner had under his care a panel patient who was suffering from an injured finger, and in the course of the treatment the finger was lanced. A few days later the patient, on the advice of his employers, went to the local infirmary, where the house surgeon again incised the finger and told the patient to return to his panel practitioner for further treatment. When, however, the latter heard that the patient had been to the infirmary he declined responsibility and refused to examine or to dress the injured part. Later the patient was attended to by some nurses, and in the end he was seen by another doctor, who took him to the infirmary again, where the finger was amputated. Now we quite agree that the panel doctor occupied a thoroughly sound position when he contended that the responsibility had been transferred from his shoulders to those of the medical officers of the infirmary. These latter had undertaken an active process of treatment, and it rested with them to see that the measures consequent on this were supplied. The case was not one in which they had acted on the approval or with the wish of the practitioner in charge of the case. On their own initiative and voluntarily they had accepted the responsibility of interference, and they could not free themselves from this by remitting the patient to another doctor. To attempt to do so must be accepted as evidence of a conception of their duty which is wholly beyond defence. Let all this be allowed, there still remains the lamentable fact that the patient was not adequately cared for, and that, whether consequential on this or not, the man lost his finger. It is therefore necessary to inquire whether the panel practitioner, in taking up an attitude of detachment and negation, adopted the course which was wisest in the circumstances. Personally we cannot come to this conclusion. We acknowledge freely that both formally and essentially the responsibility was on the infirmary doctors. But even so the patient ought not to have been allowed to suffer. To him, doubtless, the refinements of the situation were incomprehensible, and he probably felt himself entangled in a labyrinth in the construction of which he had taken no part. However defective in his appreciation of the proper method of professional procedure, he was still a suffering human being, and worthy for that reason of consideration and assistance. It was open to his medical adviser to have sent him back to the infirmary with a courteous note saying that in the circumstances it would be better for the treatment to be continued at this institution. He himself, it is true, had received no such courtesy from the official medical officer, but all the more credit would have been his due had he set so excellent an example. Again, he might have continued to treat the patient and have registered a protest in the proper quarters against the interference and discourtesy of the infirmary officials. To ask either of these things is, we recognise, to ask much. But the high claim of professional tradition to seek in all things the patient's welfare is a summons to put aside even proper pride and the sense of injustice, and to remember that the wrong-doing and negligence of our neighbours does not neutralise the most generous interpretation of our own obligations. Whatever weapons of controversy we may employ against our erring brethren, the patient himself must not suffer in the struggle. Indeed, the more certain we are of our own rectitude the less need is there to entangle other interests in the quarrel.

